# Changes of Serum Zinc-*α*2-Glycoprotein Level and Analysis of Its Related Factors in Gestational Diabetes Mellitus

**DOI:** 10.1155/2021/8879786

**Published:** 2021-02-18

**Authors:** Dongmei Xu, Jie You, Guixia Chen, Hongli Su, Li Zhang, Lingling Cui, Zhonglei Li, Guoling Huang, Caiying Feng

**Affiliations:** ^1^Department of Health, The Third Affiliated Hospital of Zhengzhou University, Zhengzhou, Henan 450052, China; ^2^Department of Clinical Nutrition, The Third Affiliated Hospital of Zhengzhou University, Zhengzhou, Henan 450052, China; ^3^Department of Obstetrics and Gynecology, Zhengzhou Central Hospital Affiliated to Zhengzhou University, Zhengzhou, Henan 450007, China; ^4^Department of Nutrition and Food Hygiene, School of Public Health, Zhengzhou University, Zhengzhou Henan 450001, China; ^5^Department of Financial, The Third Affiliated Hospital of Zhengzhou University, Zhengzhou, Henan 450052, China

## Abstract

Previous studies have discovered that zinc-*α*2-glycoprotein (ZAG) is related to insulin resistance and lipid metabolism. The aim of the study is to explore the change of serum ZAG and its related factors in gestational diabetes mellitus (GDM). Eighty newly diagnosed GDM patients were enrolled in the case group, and 80 normal pregnant women were selected as the control group. The differences of baseline data between the two groups were compared, and the change of serum ZAG level and its relationship with related indexes was analyzed. Compared to the control group, the level of serum ZAG in GDM women decreased (*P* < 0.001). What is more, the serum ZAG level of overweight and normal subjects in two groups was also found to have statistical differences. The Pearson correlation (or Spearman correlation) analysis showed that serum ZAG level was negatively correlated with FPG, FINS, HOMA-IR, and TG (all *P* < 0.05) and positively correlated with HDL (*P* < 0.05). Multiple linear regression showed that HDL and HOMA-IR were independent factors of serum ZAG (*P* < 0.05). The level of serum ZAG in patients with gestational diabetes mellitus decreased, and HDL and HOMA-IR are the influencing factors in the case group.

## 1. Introduction

Gestational diabetes mellitus is one of the common complications of pregnancy. The prevalence of GDM ranges from 1 to 18.5%, which globally has an increasing tendency year by year [1–3]. However, the etiology of GDM has not been clear yet so far, and the pathogenesis needs further study. At present, it is generally believed that maternal obesity and lipid metabolism disorder are related to the occurrence of GDM [4, 5]. Nowadays, many specialists focus their eyes on the role of adipokines, which contributes to the metabolic abnormalities in the mother. A study found statistical difference in adiponectin between GDM patients and normal pregnant women [6]. ZAG is a type of adipocytokine, secreted by adipocytes, which was first isolated and purified from human serum by Burgi et al. in 1961 [7]. It is widely found in human plasma and other various body fluids, with carrier protein, ribonuclease activity, and other functions [8, 9]. In recent years, many studies have shown that ZAG has the function of regulating immunity, cell adhesion, and melanin production [10, 11]. Some studies [12–14] also pointed out that ZAG can be used as a biomarker for the early diagnosis of cancer, and it can participate in regulating tumor cell proliferation and glucose metabolism [15]. It was also found that ZAG is not only involved in the regulation of fat metabolism in obesity but also related to the occurrence of T2DM lipid metabolism disorder [16]. A randomized controlled trial [15] found evidence to suggest that adipose tissue may be a primary source of circulating ZAG in humans and that the downregulation of ZAG expression in adipose tissue may be responsible for the reduced circulating ZAG levels in T2DM patients. In vivo and in vitro experiments also confirmed that ZAG can promote fat mobilization [17, 18]. Another study showed that ZAG was associated with downregulated lipogenic enzymes and upregulated lipolytic enzyme expressions in adipose tissue [19].

At present, most of previous research is limited to T2DM patients and other related diseases. Compared with the previous study of the relationship between ZAG and GDM, we measured the level of ZAG in the second trimester. Considering the earlier detection time, it may be more meaningful in predicting and controlling GDM. The study's aim is to explore the change of serum ZAG and its related factors in GDM, thus providing a theoretical basis for the metabolism mechanism of GDM patients. And it may provide a new direction for the prevention and treatment of GDM.

## 2. Materials and Methods

### 2.1. Subjects

All subjects of gestation age between 24 weeks and 28 weeks were recruited in the study in the Third Affiliated Hospital of Zhengzhou University from July 2018 to June 2019. All the subjects are Chinese. They all were naturally pregnant and had single pregnancy, and they did regular perinatal care and gave birth in the hospital. They undertook 75 g oral glucose tolerance test (OGTT) during their prenatal examination. Newly diagnosed gestational diabetes mellitus patients were selected as the case group. We selected normal pregnant women as controls on the same day when GDM pregnant women were diagnosed by matching the gestational week with that of the GDM pregnant women. What is more, the range of age between the GDM pregnant women and the matching controls is not more than 3 years old. And we excluded the pregnant women with chronic diseases such as diabetes, hypertension, cardiovascular and cerebrovascular diseases, severe liver and kidney diseases, tumors, mental diseases, and other pregnancy complications. Written informed consent was obtained from all participants. The study meets the requirements of the ethics committee, and the study was registered in the Chinese Clinical Trial Registry (ChiCTR2000028811).

### 2.2. Methods

The demographic and clinical characteristic data were collected, such as height, weight, body mass index (BMI), age, race, residence, systolic pressure (SBP), and diastolic pressure (DBP). Some hematologic and biochemical indexes including fasting blood glucose (FBG), blood glucose, triglyceride (TG), high-density lipoprotein (HDL), low-density lipoprotein (LDL), and total cholesterol (TC) were also collected.

The enzyme-linked immunosorbent assay (Huamei Bioengineering Co., Ltd., Wuhan, China) was used to detect the level of serum ZAG. The coefficients of variation (CV) for the intra- and interassay were lower than 8% and 10%, respectively.

ELISA kits (Huamei Bioengineering Co., Ltd., Wuhan, China) were used to detect the level of insulin, and the CV for the intra- and interassay were both lower than 15%. The insulin resistance index (HOMA-IR) was calculated and analyzed with homeostasis model assessment [20]. The calculation formula is HOMA‐IR = FPG × FINS/22.5.

### 2.3. Diagnostic Criteria for Gestational Diabetes Mellitus

According to Chinese diagnostic criteria of gestational diabetes mellitus, the critical serum glucose values of fasting and 1 hour and 2 hours after taking glucose were 5.1 mmol/L, 10.0 mmol/L, and 8.5 mmol/L, respectively. Gestational diabetes can be diagnosed if any of the three outcomes is greater than or equal to the critical value in pregnant women with fasting glucose or 75 g OGTT after 24-week gestation.

### 2.4. Statistical Analysis

The SPSS 21.0 statistical package was used to process the data of the study. Firstly, the 1-sample Kolmogorov-Smirnov test was performed to verify the normal distribution of the quantitative variables. And then normally distributed data were expressed as mean ± SD, while the quantitative data of abnormal distribution were expressed as median (25-75^th^ percentile). And qualitative data were expressed as a ratio (or percentage). The independent samples *t*-tests, Mann-Whitney *U* test, or chi-square test was used to explore the difference between the two groups. The Pearson (or Spearman) correlation analysis and multiple linear regression analysis were used to evaluate the association between the indicators. Two-tailed *P* values less than 0.05 were regarded as statistically significant.

## 3. Results

### 3.1. Comparison of Baseline Clinical and Metabolic Characteristics between the Two Groups

According to the inclusion criteria and exclusion criteria, a total of 160 subjects were enrolled in the study, with 80 subjects in each group. The age of the case group and the control group were 30.08 ± 3.43 years and 30.50 ± 3.88 years, respectively. No significant differences were found between the two groups (*P* > 0.05). As expected, there were higher levels of fasting glucose, gain weight, HbA1c, and fasting insulin in the case group, and the difference between the two groups was statistically significant (*P* < 0.05). HOMA-IR in the case group has higher level than that in the control group, and the difference was statistically significant (*P* < 0.001). We also investigated the level of serum lipid in the two groups, and the results showed that the case group has higher level of TG and lower level of HDL, and the difference was significant (*P* < 0.05). However, there was no statistical difference in TC and LDL between the two groups (*P* > 0.05). The other baseline clinical and metabolic characteristics such as height, SBP, and SDP showed no statistical difference in the two groups (*P* > 0.05), as shown in Table 1.

### 3.2. Comparison of Serum ZAG Levels between the Case Group and Control Group

**Figure 1:**
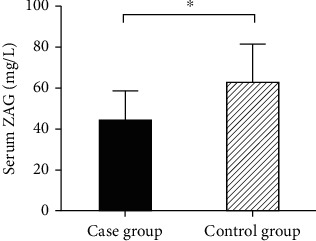
Comparison of serum ZAG between the two groups.

**Figure 2:**
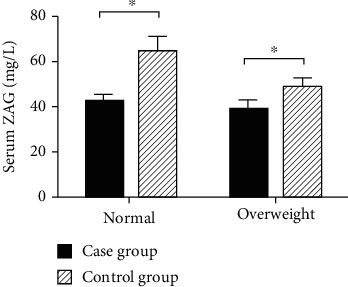
Comparison of serum ZAG levels between the overweight and normal groups in the case group and control group.

Compared to the control group, the serum ZAG level in the case group was decreased, and the difference was statistically significant (43.94 ± 14.51 mg/L vs. 62.57 ± 19.05 mg/L, *P* < 0.001, Figure 1). With BMI = 25.0 kg/m^2^ as the cut-off point, we divided the case group and the control group into the normal group (BMI < 25.0 kg/m^2^) and overweight group (BMI ≥ 25.0 kg/m^2^). The ZAG levels of the normal group were 41.23 ± 2.15 mg/L in the case group and 64.06 ± 4.27 mg/L in the control group, while the ZAG levels of the overweight group were 39.83 ± 3.08 mg/L in the case group and 48.69 ± 2.97 mg/L in the control group. We found that the level of serum ZAG was declined in both the overweight group and the normal group, and the differences were statistically significant (*P* < 0.05), as shown in Figure 2.

### 3.3. Correlation Analysis of Serum ZAG and Related Factors in the Case Group

With ZAG as the dependent variable and other clinical indicators as independent variables, the correlation analysis was conducted. The results are shown in Table 2. It showed that serum ZAG levels of pregnant women in the case group were negatively correlated with FPG, FINS, HOMA-IR, and TG (*r* = −0.416, -0.167, -0.236, and -0.328, *P* < 0.05). And the results also showed that serum ZAG was positively correlated with HDL (*r* = 0.279, *P* = 0.012). However, no relationship was found between the serum ZAG and age, BMI, fat mass, etc. (*P* > 0.05).

### 3.4. Multiple Linear Regression Analysis of Serum ZAG in the Case Group

FPG, FINS, HOMA-IR, TG, and HDL were included in the regression model as independent variables, and the results showed that HOMA-IR (*β* = −3.168, *P* < 0.05) and HDL (*β* = 2.551, *P* < 0.05) were independent influencing factors of serum ZAG. When conducting the multiple linear regression analysis, stepwise regression was used. What is more, the inclusion criterion was *P* ≤ 0.05 and the exclusion criterion was *P* ≥ 0.1.

## 4. Discussion

According to the study, we found that the level of serum ZAG level decreased in GDM patients, compared to the control group. And subgroup analysis showed that the GDM patients also tended to have lower ZAG levels in both overweight pregnant woman and normal subjects. Finally, the regression analysis showed that HOMA-IR and HDL were independent influencing factors of serum ZAG level in GDM patients. But whether serum ZAG has a certain role in the metabolism of serum glucose and lipid in GDM patients still needs further study. Some experts have done some research. Yang et al. [15] found that ZAG was related to insulin resistance, which was consistent with the view. Naf et al. [6] also pointed out that serum ZAG level in GDM patients was related to HDL, suggesting that ZAG was involved in lipid metabolism in GDM patients. However, no statistical difference was found between the GDM group and the NGT group in serum ZAG level, which may be caused by factors such as different races, research methods, and sample size. In addition, Naf et al. measured the serum ZAG level before delivery, while our study collected the detection indicators of pregnant women with GDM at 24 to 28 weeks, which may lead to the difference between the two studies.

In summary, the lower levels of serum ZAG might relate to HOMA-IR and HDL. Serum ZAG might play a certain role in lipid metabolism and insulin resistance in patients with GDM. Some experts found that ZAG could reduce adiponectin, insulin receptor substrate-1, and glucosetransporter-4 gene expression in primary human adipocytes, which indicates that ZAG might play an important role in modulating whole-body and adipose tissue insulin sensitivity [21]. Currently, there are few studies on the relationship between serum ZAG and GDM, and more evidence-based medical evidence is still needed. The potential limitations of our study are the relatively small sample and the geographical distribution. Therefore, a multicenter and multiarea large-sample prospective study is still needed to further clarify the relationship between serum ZAG and GDM.

## Figures and Tables

**Table 1:** The clinical and metabolic characteristics of the two groups.

Characteristics	Case group (*N* = 80)	Control group (*N* = 80)	t/Z	*P*
Age (years)	30.08 ± 3.43	30.50 ± 3.88	-0.734	0.464
Height (cm)	161.86 ± 4.71	161.35 ± 4.40	-0.740	0.460
Weight (kg)	62.73 ± 10.65	58.18 ± 7.18	-3.168	0.002
SBP (mmHg)	114.21 ± 6.90	113.26 ± 5.41	-0.970	0.334
DBP (mmHg)	68.16 ± 7.36	67.50 ± 5.84	-0.621	0.535
BMI (kg/m^2^)	24.58 ± 3.93	22.96 ± 2.82	-3.112	0.002
Gain weight (kg)	6.91 ± 2.14	5.32 ± 1.23	-2.474	0.015
HbA1c (%)	5.74 ± 0.45	4.81 ± 0.33	-14.906	<0.001
Fat mass (kg)	20.8 (16.93, 22.52)^∗^	20.3 (15.35, 21.98)^∗^	-2.273	0.023
Fat (%)	34.47 ± 6.01	33.23 ± 4.63	-1.521	0.130
FPG (mmol/L)	5.03 ± 0.60	4.45 ± 0.35	-7.817	<0.001
OGTT-1 h	9.49 ± 1.68	6.92 ± 1.35	-10.723	<0.001
OGTT-2 h	8.44 ± 1.55	6.40 ± 1.00	-9.907	<0.001
FINS (mU/L)	7.56 (5.69, 14.4)^∗^	4.92 (4.01, 10.44)^∗^	-4.363	<0.001
HOMA-IR	1.59 (1.28, 3.22)^∗^	0.94 (0.79, 1.81)^∗^	-5.453	<0.001
TC (mmol/L)	5.51 ± 1.32	5.59 ± 1.87	0.293	0.770
TG (mmol/L)	2.83 (2.21, 3.48)^∗^	2.58 (1.89, 3.36)^∗^	-7.152	<0.001
HDL (mmol/L)	1.73 ± 0.47	2.07 ± 0.62	3.943	<0.001
LDL (mmol/L)	3.06 ± 0.69	2.86 ± 0.86	-1.641	0.103
Residence			1.604	0.205
City, *n* (%)	48 (60.00)	55 (68.75)		
Rural, *n* (%)	32 (40.00)	25 (31.25)		

SBP: systolic pressure; DBP: diastolic pressure; BMI: body mass index; HbA1c: glycosylated hemoglobin; fat (%): percent of body fat; FPG: fasting blood glucose; TG: triglyceride; HDL: high-density lipoprotein; LDL: low-density lipoprotein; TC: total cholesterol; ZAG: zinc-*α*2-glycoprotein. ^∗^Value data are presented as median (25-75^th^ percentile), and others are presented as mean ± SD.

**Table 2:** Correlation between serum ZAG and related indexes in the case group.

	Age	BMI	Fat mass	Fat (%)	FPG	FINS	HOMA-IR	TC	TG	HDL	LDL
*r*	0.089	-0.219	-0.015	-0.086	-0.416	-0.167	-0.236	0.018	-0.328	0.279	0.153
*P*	0.432	0.051	0.895	0.450	<0.001	0.035	0.015	0.876	<0.001	0.012	0.174

## Data Availability

The figures and tables used to support the findings of this study are included within the article.
